# Anterior Chamber Characteristics, Endothelial Parameters, and Corneal Densitometry After Descemet Stripping Automated Endothelial Keratoplasty in Patients With Fuchs Dystrophy

**DOI:** 10.18502/jovr.v16i2.9078

**Published:** 2021-04-29

**Authors:** Remzi Karadag, Kristin M. Hammersmith, Parveen K. Nagra, Christopher J. Rapuano

**Affiliations:** ^1^Veni Vidi Eye Center, Istanbul, Turkey; ^2^Cornea Service, Wills Eye Hospital, Sidney Kimmel Medical College at Thomas Jefferson University, Philadelphia, USA

**Keywords:** Anterior Chamber Parameters, Corneal Densitometry, Corneal Thickness, Descemet Stripping Automated Endothelial Keratoplasty, DSAEK, Endothelial Cell, Fuchs Dystrophy

## Abstract

**Purpose:**

To compare anterior segment parameters in patients with Fuchs endothelial dystrophy (FED) who underwent Descemet stripping automated endothelial keratoplasty (DSAEK) in one eye and no corneal surgery in the fellow eye.

**Methods:**

This prospective study was conducted on 28 eyes of 14 patients with FED who underwent DSAEK in one eye at least one year prior (DSAEK group) and no corneal surgery in the fellow eye (control group). Each eye was analyzed with the anterior segment optical coherence tomography, specular microscopy, and Scheimpflug imaging systems. Data were compared between the two groups.

**Results:**

The mean age of the patients was 76.9 ± 7.0 years. There were no statistically significant differences in the mean central corneal thickness (CCT), central anterior chamber depth, anterior chamber angle parameters, cylinder and keratometry values between two groups (all *P*-values > 0.05). The paracentral corneal thickness, corneal volume, endothelial cell density, and hexagonal cell ratio measurements were statistically significantly higher in the DSAEK group than the control (all *P*-values < 0.05), and anterior chamber volume in the DSAEK group was significantly less than the control (*P* = 0.046). While posterior and total corneal densitometry values in the DSAEK group were statistically significantly lower than the control (*P* < 0.001 and *P* = 0.011, respectively), there were no statistically significant differences in the anterior or middle corneal densities (*P *= 0.108 and *P* = 0.134, respectively).

**Conclusion:**

We found that total corneal densitometry value decreased in DSAEK group. Although DSAEK surgery did not affect the anterior chamber angle parameters, it reduced the anterior chamber volume and increased the corneal volume and paracentral corneal thickness due to the addition of the DSAEK graft.

##  INTRODUCTION

Fuchs endothelial dystrophy (FED) is a fairly common corneal disorder that can result in visual impairment and require corneal transplantation.^[1]^ Because the pathology is primarily localized to the posterior corneal layers including Descemet membrane and endothelium, posterior lamellar keratoplasty is the treatment of choice for patients with FED. Descemet stripping automated endothelial keratoplasty (DSAEK) is one of the most commonly performed posterior lamellar surgeries for FED. This method involves the replacement of the posterior diseased part of the cornea with donor endothelium, Descemet membrane, and a small amount of stroma.^[2]^ This technique has a number of advantages over penetrating keratoplasty (PK), including less induced astigmatism, fewer suture-related complications, fewer high-order aberrations, faster visual rehabilitation, and a stronger wound.^[2,3]^ In the literature, a few studies have investigated biomechanical properties and keratometry of cornea, and some anterior segment (AS) parameters after the DSAEK surgery in patients with FED.^[4,4,5,6,7]^ Some studies have shown that DSAEK leads to a small hyperopic shift,^[4,5]^ residual corneal aberrations, glare and reduced contrast sensitivity compared to normal.^[6,7]^ To the best of our knowledge, no studies have characterized the anterior chamber and corneal parameters using three different devices after DSAEK surgery in one eye in patients with FED.

The aim of this study was to compare anterior chamber and corneal parameters after DSAEK in one eye and no corneal surgery in the other eye in patients with FED.

##  METHODS

This intra-subject comparative study was performed at the Wills Eye Hospital Cornea Service, Philadelphia, PA. Ethics committee approval was obtained from the Wills Eye Hospital Institutional Review Board. A written informed consent was obtained from all participants before entering the study. This study was conducted on 28 eyes of 14 patients with the diagnosis of FED who underwent DSAEK in one eye at least one year prior (DSAEK group) and no corneal surgery in the fellow eye of the same patient (control group). Patients with other ocular comorbidities besides FED and a history of cataract surgery were not included. We only accepted bilateral pseudophakic patients with posterior chamber intraocular lenses in order to standardize the two groups.

In addition to routine ophthalmic testing, additional measurements were obtained by one examiner (RK) using the following three imaging systems. Each subject had both eyes imagined with the Visante TD-OCT (Carl Zeiss Meditec, Inc., Dublin, California, USA), the Pentacam HR Scheimpflug imaging system (Pentacam HR, Oculus, Wetzlar, Germany), and specular microscopy (Konan Non-contact Specular Microscope, Noncon Robo-CA, Konan Medical Inc, Hyogo, Japan).

**Figure 1:**
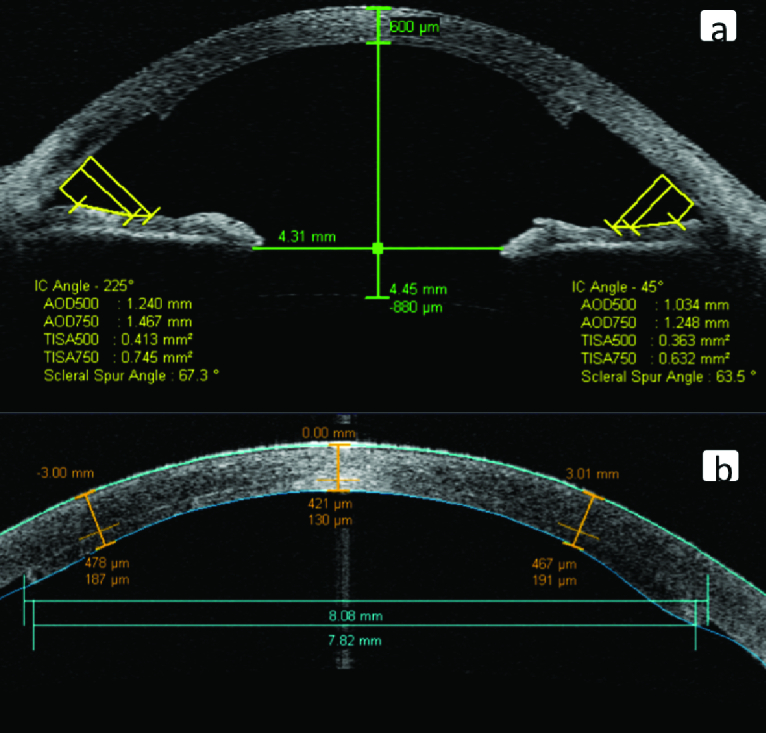
(a) Anterior chamber angle parameters were measured by AS-OCT. (b) Anterior chamber depth, corneal thickness, and graft thickness.

The measurements obtained with AS-OCT included paracentral corneal thickness (3 mm from the corneal center), anterior chamber depth, DSAEK graft diameters, scleral spur (SS) angle, angle opening distance (AOD), and trabecular-iris-space area (TISA) in eight different angles meridians (0, 45, 90, 135, 180, 225, 270, and 315 degree angles). SS-based measurements at all angles were measured with the internal calculation tool of the Visante device [Figure 1a]. The AOD500 and AOD750 were calculated as the distance between the trabecular meshwork and the iris 500 and 750 μm anterior from the SS, respectively. TISA 500 and TISA750 space area are defined as the area between the inner cornea-scleral wall and iris surface, which measured 500 and 750 μm anterior from SS, respectively. The calculation of AOD and TISA values using the internal Visante tool is demonstrated in Figure 1. Because all measurements were obtained only at 0 and 180 degrees in all patients, 0 and 180 degree values were used for statistical analysis for SS angle, AOD, and TISA values. Four measurements were taken for central corneal thickness (CCT), central donor graft thickness, and anterior chamber depth, and the mean of the four measurements were used for statistical analysis [Figure 1b]. Corneal lamellar thickness measurements for DSAEK graft and recipient cornea were obtained using the measurement caliper provided by the software in all eight meridians and centrally. The corneal side of graft diameter and the anterior chamber side of graft diameter were measured, defined as the straight-line distance between the anterior edges and the posterior edges of the graft, respectively [Figure 1b].

**Figure 2:**
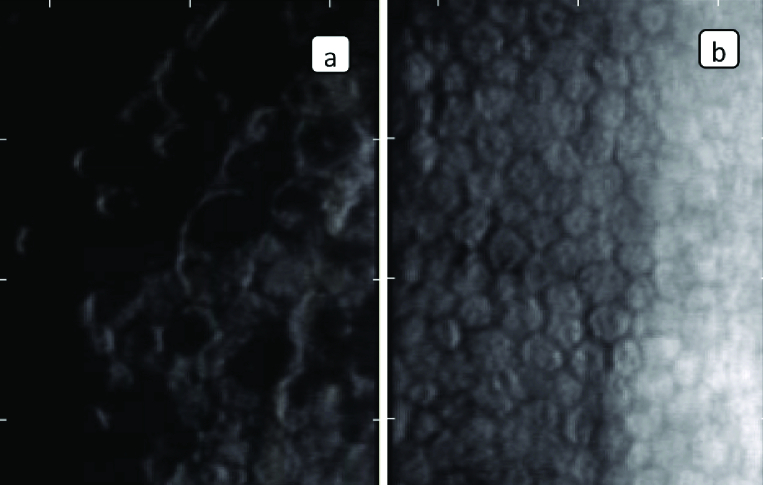
(a) Endothelial cells parameters in eye with FED. (b) Endothelial cells parameters in DSAEK.

Specular microscopy was used to measure endothelial cell density (ECD) and hexagonal cell ratio [Figures 2a and 2b].

Keratometry, cylinder, anterior chamber volume, corneal volume (CV), and corneal clearance were measured using the Pentacam Scheimpflug imaging system. Pentacam software module enables a standardized corneal densitometry analysis to be performed. We used anterior, central, posterior, and total corneal layers densitometry measurements in the central 4 mm zone of the cornea for the statistical analysis.

Statistical analysis was performed with analysis software (Statistical Package for Social Science, version 16; SPSS Inc., Chicago, IL). Comparison of measurements between two groups was analyzed by student *t*-test. *P* < 0.05 was considered statistically significant.

##  RESULTS

We included six female and eight male patients. The mean age was 76.9 ± 7.0 years. DSAEK surgery was performed in nine right and five left eyes. The mean time after DSAEK was 28.1 ± 9.2 months. The mean corneal side of graft diameter was 8.16 ± 0.26 mm and the mean anterior chamber side of graft diameter was 7.80 ± 0.27 mm [Table 1]. Patients' demographics are shown in Table 1.

AS-OCT results are shown in Tables 2 and 3. There were no statistically significant differences in mean CCT value between the two groups (*P* = 0.253). Mean paracentral corneal thickness measurements were found statistically significantly higher in the DSAEK group than the control group in all eight meridians (*P*-values < 0.05) [Table 2]. Although mean anterior chamber depth values were lower in the DSEAK group than the controls, there was no statistically significant difference between groups (*P* = 0.32). There were also no statistically significant differences between groups according to AOD500, AOD750, TISA500, TISA 750, and SS angle measurements in 0- and 180-degree meridians (*P*-values > 0.05) [Table 3]. ECD and hexagonal cell ratio values measured by specular microscopy were found statistically significantly higher in the DSAEK group than the controls (*P* < 0.001 and *P* = 0.001, respectively) [Table 1].

There were no statistically significant differences in the cylinder and keratometry values measured by Pentacam Scheimpflug imaging (*P*-values > 0.05). The mean CV in the DSAEK group was statistically significantly higher than the control group (*P* < 0.001) and the anterior chamber volume in the DSAEK group was statistically significantly lower than the control group (*P* = 0.046). When the groups were compared in terms of corneal densitometry measurements, the posterior and total corneal densitometry values in the DSAEK group were statistically significantly lower than the control (*P* < 0.001 and *P* = 0.011, respectively). Although there were lower values in the anterior or middle corneal densitometry in DSAEK group, the differences were not statistically significant (*P* = 0.108 and *P* = 0.134, respectively) [Table 1].

##  DISCUSSION

Fuchs endothelial dystrophy is a common indication for keratoplasty in developed countries. Pathologic findings of FED are localized to Descemet's membrane and endothelial cells. Therefore, replacement of only the endothelial cells and Descemet's membrane should improve the symptoms and findings of the disease.^[2,8]^

DSAEK is one of the posterior lamellar surgeries. Although, it has several advantages over PK, it suffers some drawbacks. One of the disadvantages of DSAEK is a high surgically induced loss of endothelial cells. Current long-term endothelial cell studies have shown a slowing of the endothelial cell loss as compared with PK after the first year.^[9,10,11,12]^ In the current study, when we compared ECD between the eye with DSAEK and the fellow eye with no corneal surgery, we found that the ECD was significantly higher in eyes with DSAEK than the fellow eye with FED.

Corneal transparency has been evaluated using the Pentacam Scheimpflug system, which has the capability to measure light scattering and corneal haze by densitometry.^[5,13]^ The corneal epithelial cell layer and the corneal endothelium are the major origins of light scattering while the corneal stroma retains low scattering property.^[14]^ Deterioration of the collagen matrix due to edema in FED and subsequent corneal scarring can cause an increase in the light scattering and result in glare. Arnalich-Montiel et al demonstrated that densitometry values after DSAEK were significantly higher than in normal subjects for full thickness, posterior and anterior parts of the paracentral cornea, and for the anterior part of the central cornea.^[15]^ They reported that their results could depend on the optical effect of the DSAEK interface. Similarly, Baratz and colleagues suggested that glare might be caused by scarring at the lamellar graft–host interface or by abnormalities in the residual anterior host cornea in DSAEK.^[16]^ In the present study, when the groups were compared in terms of corneal densitometry measurements, we found the posterior and total corneal densitometry values in the DSAEK group were statistically significantly lower than the control eye with FED. Although there were lower corneal densitometry values in the anterior or middle layers in the DSAEK group, the differences were not statistically significant. It was demonstrated that DSAEK surgery was associated with improved corneal densitometry when compared with the fellow eye with FED, most likely due to the lack of corneal edema.

Bahar et al^[17]^ showed that anterior corneal astigmatism, anterior chamber angle, anterior chamber depth, and anterior chamber volume values measured by Pentacam did not change significantly following DSAEK surgery, although CCT value was significantly decreased and CV value and posterior corneal astigmatism were significantly increased. They did not give any information regarding the graft thickness they used in their study.^[17]^ In the present study, we noticed that mean CV value was significantly higher in eyes with DSAEK than the fellow eyes with FED. We also found that the mean central graft thickness was 145 μm. We believe that the increase in CV probably represents the replacement of the recipient's Descemet membrane and endothelium by the donor's lenticule which contains some donor stroma; this was greater than the decrease in corneal edema in eyes with DSAEK. Additionally, we measured DSAEK graft diameters (corneal and anterior chamber side) in this study. We did not find any correlation among graft diameters and the others anterior chamber parameters.

Terry et al reported that preoperative DSAEK graft thickness might have a small effect on visual outcomes in the extremes of thickness, but not in the common range of 100–200 μm.^[18]^

There is only one study in the literature evaluating angle parameters after DSAEK surgery in patients with Fuchs.^[17]^ The Pentacam Scheimpflug device was used in that study. Bahar et al^[17]^ only measured the anterior chamber angle as the anterior chamber angle parameter. They found that DSEAK did not significantly change the anterior chamber angle. In our study, we investigated multiple anterior chamber angle parameters (angle degree, AOD500, AOD750, TISA 500, and TISA 750). Unlike the Bahar et al study,^[17]^ we used an AS-OCT device instead of Pentacam Scheimpflug imaging for these measurements. In our study, there were no statistically significant differences between groups according to AOD500, AOD750, TISA500, TISA 750, and SS angle measurements.

In DSAEK, only the posterior lamella is changed as compared to full-thickness PKP that gives it the benefit of faster visual improvement. Suture-related problems especially astigmatism is also reduced. DSAEK is associated with a hyperopic shift of the order of approximately 0.8–1.5 D, depending on the lenticule thickness being transplanted. Hence, the intraocular lens power needs to be refined according to this phenomenon while doing DSEAK combined with cataract surgery. Unexpected refractive errors may sometimes occur in some patients who have undergone DSAEK and cataract surgery.^[19]^ Piggy-back intraocular lenses may be needed for the treatment of these patients. As seen in this study, it should be kept in mind that the anterior chamber depth and volume may be affected by DSEAK surgery in such patients and measurements should be made accordingly.

In conclusion, we found that total corneal densitometry value decreased in the DSAEK group. Although DSAEK surgery does not affect the anterior chamber angle parameters, it decreases the anterior chamber volume and increases the CV and paracentral corneal thickness due to the DSAEK graft thickness. We believe that due to the reduced anterior chamber volume, the anterior chamber lenses should be used with caution in these patients.

##  Financial Support and Sponsorship

Nil.

##  Conflicts of Interest

There is no conflict of interest.
